# Local and Systemic Pathogenesis and Consequences of Regimen-Induced Inflammatory Responses in Patients with Head and Neck Cancer Receiving Chemoradiation

**DOI:** 10.1155/2014/518261

**Published:** 2014-03-16

**Authors:** Elvio G. Russi, Judith E. Raber-Durlacher, Stephen T. Sonis

**Affiliations:** ^1^Department of Radiation Oncology, University Teaching Hospital A.O. “S. Croce e Carle”, Via M. Coppino 26, 12100 Cuneo, Italy; ^2^Department of Oral and Maxillofacial Surgery, Academic Medical Center, University of Amsterdam, Gustav Mahlerlaan 3004, 1081 LA Amsterdam, The Netherlands; ^3^Division of Oral Medicine, Brigham and Women's Hospital and the Dana-Farber Cancer Institute and Biomodels, LLC, 75 Francis Street, Boston, MA 02115, USA

## Abstract

Treatment-related toxicities are common among patients with head and neck cancer, leading to poor clinical outcomes, reduced quality of life, and increased use of healthcare resources. Over the last decade, much has been learned about the pathogenesis of cancer regimen-related toxicities. Historically, toxicities were separated into those associated with tissue injury and those with behavioural or systemic changes. However, it is now clear that tissue-specific damage such as mucositis, dermatitis, or fibrosis is no longer the sole consequence of direct clonogenic cell death, and a relationship between toxicities that results in their presentation as symptom clusters has been documented and attributed to a common underlying pathobiology. In addition, the finding that patients commonly develop toxicities representing tissue injury outside radiation fields and side effects such as fatigue or cognitive dysfunction suggests the generation of systemic as well as local mediators. As a consequence, it might be appropriate to consider toxicity syndromes, rather than the traditional approach, in which each side effect was considered as an autonomous entity. In this paper, we propose a biologically based explanation which forms the basis for the diverse constellation of toxicities seen in response to current regimens used to treat cancers of the head and neck.

## 1. Introduction

The past decade has seen major shifts in how we view the biology and consequences of regimen-related toxicities associated with cancer therapy. Tissue-specific damage such as mucositis, dermatitis, or fibrosis is no longer thought to be the sole consequence of direct clonogenic cell death. A relationship between toxicities that results in their presentation as symptom clusters has been documented and attributed to common underlying pathobiology [1, 2]. Active roles for the local microbiota and tumour as biologically active contributors and modifiers of toxicity development are being assessed. Genomic differences among patients have been identified which are major determinants of toxicity risk [3–5]. And the finding that patients commonly develop toxicities representing tissue injury (i.e., diarrhoea) outside radiation fields and side effects such as fatigue or cognitive dysfunction suggests the generation of systemic and local mediators. Cumulatively, this information has formed the basis for a robust pipeline of investigative agents that offer the hope of effective toxicity interventions.

Among cancer patients, those being treated for cancers of the head and neck (HNCPs) represent one of the most robust populations to evaluate and analyse focal tissue injury such as mucositis or dermatitis or systemic side effects such as fatigue, cachexia, or cognitive dysfunctions [6–9]. Furthermore, rarely do patients have a single toxicity. Rather, treatment-related complications appear to occur as nonrandom clusters [1, 2], which share a common underlying pathobiological basis. In fact, it might be most appropriate to consider the study of “toxicity syndromes,” rather than the traditional approach in which each side effect was considered as an autonomous entity.

The historical reductionist view that attributed iatrogenic damage solely to the clonogenic cell death of tissue stem cells, mostly in the epithelium of the entire gastrointestinal tract, has been experimentally reevaluated to reveal that iatrogenic toxicities including mucositis [10, 11], dermatitis [12], and pneumonitis [13, 14] represent the culmination of a series of biologically complex events that occur in all directly and indirectly injured tissues [15]. In addition, the observation of toxicities, which are systemically manifested, have provided a rationale for the application of the abscopal effect to normal tissues in addition to tumours [16]. This hypothesis suggests that focal radiation, even in the absence of concomitant chemotherapy (CT), can result in biologically active mediators that have diffuse targets at remote sites. The clustering of CT- and radiotherapy (RT)-induced toxicities sharing common pathobiology reported by Aprile et al. [1] provided a biological basis for clinical observations. Understanding the nature of the genesis of these toxicities is critical to establishing an effective interventional strategy. Based on the pathobiology of diseases which result in similar phenotypes (i.e., chronic fatigue syndrome, Crohn's disease, and Sjogren's syndrome as examples), it seems that inflammatory pathways and mediators are likely candidates for this role.

It is becoming increasingly clear that the wide variety of proteins elicited by CT or RT to cause local toxicities may have significant abscopal effects that put the patient at risk for a systemic inflammatory reaction that, in many ways, resembles what is seen clinically in sepsis. While the true definition of sepsis is not satisfied in cases with no identifiable microbial invasion, the scope of a systemic inflammatory response can result in marked clinical morbidity or even death [17–21]. Moreover, bacteraemia, due to the loss of integrity of a physical barrier (mucosa, skin), are not always associated with sepsis [22, 23] but may lead to sepsis when they are associated with a panoply of nonspecific inflammatory responses [24].

In addition, it has been postulated that an inflammatory response induced by the tumour itself [25] may play a role and, together with inflammation induced by iatrogenic cytolysis, may contribute to the main adverse events in CT-RT-treated HNCPs.

In this manuscript, we attempt to develop a biologically based explanation which forms the basis for the diverse constellation of toxicities seen in response to current regimens used to treat cancers of the head and neck.

## 2. Mucosal Injury

Until the late 1990's [26], the historical paradigm of cancer regimen-related epithelial injury held that damage was essentially the consequence of nonspecific clonogenic cell death in which rapidly dividing epithelial basal cells were indiscriminately destroyed by chemotherapy or radiation. This hypothesis was subsequently overturned in favour of a concept which identified that radiation and chemotherapy induce a plethora of biological events, largely in the submucosa, which activate a collection pathways which in turn mediate basal cell injury and death [27, 28]. A role for the local mucosal environment, including microorganisms, has also been suggested [29] but is still in need of study as interventional strategies aimed at eliminating or modifying the microbial flora have failed [30–33]. Furthermore, it is possible that rather than being inert to the effects of radiation, the functional characteristics of the gastrointestinal tract microflora might be inadvertent targets of radiation and, when radiated, undergo changes that modify their potential effects on tissue [34–36].

The evolution of CT-RT-induced mucosal injury was schematically classified in a five-phase model by Sonis [28]: initiation, upregulation/activation, signal amplification, ulceration, and healing. Interestingly, the activation of at least 14 canonical pathways has been identified as a consequence of chemoradiation (CRT). Thus, the “engine” which drives tissue and systemic symptom development is diverse.

### 2.1. Oxidative Stress and the Innate Immune Response as Initiators

The initiation of RT or CT-induced tissue injury is associated with biological events, clonogenic cell death as a consequence of DNA damage and strand breaks [37], oxidative stress, and activation of the innate immune response. Oxidative stress results in the creation of reactive oxygen species (ROS) inside injured cells [15, 29] at a rate that overcomes cell repairing capability [3–5].

ROS created by the ionization of intracellular water [38] cause a spectrum of lesions in cellular macromolecules (e.g., lipid peroxidation) [39]. These macromolecular lesions can damage intracellular organelles such as mitochondria [40, 41], which in turn release additional ROS. ROS further damage cell membranes and connective tissue, stimulate macrophages, and trigger a cascade of critical biological molecules that activate the immune inflammatory response [39, 42–47]. Nuclear factor erythroid 2-related factor-2 (Nrf2) has been implicated as an important element in mediating oxidative stress and preclinical data suggest that it may be a relevant target for toxicity intervention [48, 49].

Data supporting a role for the innate immune response in the genesis of radiation injury continues to accumulate. It also seems highly probable that normal cells, made apoptotic or necrotic by CT or RT [50], release endogenous damage-associated molecular patterns (DAMPs) [51] or chemoradiation associated molecular patterns (CRAMPs) [15], which play an integral role in initiating inflammation toxicity. An example of CRAMPs is the high-mobility group box 1 (HMGB1) [52]. In healthy cells, HMGB1 is located in the nucleus, where it facilitates DNA assembly. This molecule is released by necrotic cell death and pulsatile-released by cells made apoptotic by RT and CT [50]. Once outside the cell, HMGB1 has the potential to activate the host's immune system [15, 53] via the activation of the multiple surface receptors including Toll like Receptor (TLR)2, TLR4, and Receptor for Advanced Glycation End products (RAGE) [54].

The true biological and clinical consequences of HMGB1 [55, 56] are unresolved. The observation that high HMGB1 serum levels were associated with increased risk of sepsis-mediated death might be associated with the effectiveness of the molecule as a potent mediator of a systemic inflammatory response. Somewhat perplexing is the observation that HMGB1 activation is more strongly associated with low radiation exposure [57]. In experimental settings, HMGB1 was found to be involved in the loss of endothelial barrier function [58], the increase of both ileal mucosal and alveolar permeability [59–61], and the fostering of bacterial translocation to mesenteric lymph nodes [58, 60].

Table 1 summarises the factors hypothesized to be involved in the initiation of the pathobiology of mucositis and other RT-CT associated toxicities sharing the involvement of inflammation mediators. These factors can cause injury to cells, but not of sufficient magnitude from a toxicity standpoint to explain the extensive injury that characterizes the clinical presentation of mucositis [37].

Activation of the biological cascade within minutes [10] of RT and, long before any tissue changes are noted, changes in gene expression are manifest by a diverse group of cells within the targeted tissue including macrophages, endothelial cells, and fibroblasts. A range of genes with diverse functional ramifications is expressed. Whether this is a consequence of damage to cells or, rather, a consequence of the immune system itself needs to be confirmed [15, 99]. However, the involvement of pattern recognition receptors (PRRs) [100] such as TLR [101] and RAGE receptors [102] of the host's innate immune system has been hypothesized. Criswell et al. [103] demonstrated the activation of transcription factors such as NF-**κ**B, p53, and SP1 related retinoblastoma, ceramide pathway, and Nrf2 transcription factors [48, 49] and their role in the development of radiation-induced mucosal injury has been confirmed by others. At least, 14 canonical pathways associated with the development of CT-RT mucositis are triggered [11]. NF-**κ**B seems to have a central-hub role in activating inflammation [28, 53, 86, 104]; its activation precedes peaks in proinflammatory cytokines in mucosa after CT and upregulates cyclooxygenase-2 (COX-2) [105, 106] in submucosal fibroblasts and endothelial cells after radiation.

Disruption of connective tissue fibronectin leads to the deregulation of matrix metalloproteinases (MMP) [81, 107] which impact tissue injury and inflammation. In animal model studies, a significant alteration in both gene expression-tissue levels of MMPs and tissue inhibitor of metalloproteinase (TIMPs) following CT was found to be correlated with histopathological alterations [108].

Finally, the activation of the peripheral nervous system via pain fibres is conceivable. The presence of receptors (e.g., TLR4 and CD14) for inflammatory products on nociceptive fibres has been shown [73, 109]. The activation of peripheral nerve endings via these receptors [74] ultimately results in the local release of several neurotransmitters and neuropeptides (substance P and calcitonin gene-related peptide 1 (CGRP1)), which have strong vasodilatory and chemotactic properties [110]. This may explain the reversible pruritus and faint erythema that develop during the first hours after irradiation [75, 76, 111].

### 2.2. Signal Amplification and Feedback

#### 2.2.1. Local Effects: Intracellular and Intercellular Signalling Loops

Many of the proteins produced during the primary damage response (upregulation phase) also amplify proinflammatory pathways which intensifies primary damage [28]. Accordingly, a broad range of biologically active proteins accumulates and targets the mucosal tissues (endothelial, epithelial, mesenchymal, and neuronal endings) triggering intracellular and intercellular feedback mechanisms. These feedback mechanisms may induce “vicious circles,” which can also involve distant organs via neuronal and bloodstream networks [86].

Below, we present examples of interrelated feedback mechanisms that may act alone or in concert and are thought to play a role in the pathobiology of tissue-centric toxicities such as mucositis and dermatitis.

*Activation of Transcription Factors (NF-*κ*B)-Cytokines (e.g., TNF-*α*) Transcription Factors (NF-*κ*B) Loop*. NF-*κ*B, upregulated in the previous phase, acts as a “gatekeeper” for various inflammatory pathways one of which is the proinflammatory pathway characterized by cytokines [10] such as TNF, IL-6 [112, 113], and IL-1*β* [12, 87]. In turn, these cytokines (particularly TNF-*α*) are potent activators of NF-*κ*B [10], sphingomyelinase [114–116], and members of the TNF receptor family [46]. These loops drive the NF-*κ*B and ceramide pathways to produce and accelerate tissue injury and initiate mitogen-activated protein kinase (MAPK) signalling. MAPK signalling then activates c-JUN aminoterminal kinase [117], which plays a role in regulating the AP1 transcription factor, which, in turn, is thought to affect MMP secretion [82, 107, 108].

In addition, NF-*κ*B upregulates cyclooxygenase-2 (COX-2), an inducible enzyme involved in inflammation, through its role in prostaglandin production [106, 118]. COX-2 expression parallels with the development of ulcerative mucositis.

Coupled with proinflammatory cytokines, it is now apparent that anti-inflammatory cytokines such as IL-10 [119], IL-11 [120, 121] and the anti-inflammatory amino acid decapeptide (RDP58) [122] play a key inflammatory role in both oral and GI RT/CT toxicities [112, 121, 123–126].

The activation of this “cytokine storm” favours the chemoattraction of immune cells (mononuclear cells and macrophages) causing local infiltration and oedema.

The importance of inflammatory pathways in regimen-related mucosal injury has potential therapeutic leverage. Interventions aimed at attenuating proinflammatory cytokines or stimulating anti-inflammatory cytokines may offer a way to prevent toxicities [120, 121, 127–131].

*Peripheral Neuronal Amplification Loop.* While a role of neurotransmitters and neuropeptides in mucosal injury has not been well studied, it is quite possible that the activation of peripheral nerve endings and the release of several neurotransmitters and neuropeptides (substance P and calcitonin gene-related peptide *α* (CGRP1)) lead to the recruitment of innate immune cells. Studies of genetic predilection for regimen-related toxicities demonstrate the presence of single nucleotide polymorphisms associated with genes for both substance P and CGRP1 [132]. These mechanisms could potentiate the local immune response and thereby lead to the activation of additional nociceptive sensory nerves in a positive feedback manner [77].

*Inflammation-Coagulation Loop.* Endothelial damage and iatrogenic-induced cytokine synthesis upregulate procoagulation [79, 80, 133]. Indeed, laboratory and* in vivo* studies showed the potential of upregulating coagulation “tissue factor” (TF) by means of inflammatory mediators such as TNF-*α* [134], C-reactive protein (C-RP) [135], and long pentraxin-3 [136] (PTX3). In turn, TF generates coagulant mediators (FVIIa, FXa, FIIa, and Fibrin) that upregulate inflammatory mediators by means of protease-activated receptors (PARs) and TLR-4 [137–139].

This triggers an inflammation-coagulation circuit that increases local and/or systemic proinflammatory and procoagulative activity [137, 139]. The potential therapeutic implications of this loop is illustrated by the finding that hirudin, a direct thrombin inhibitor, was observed to ameliorate radiation induced intestinal toxicity in a rat model [140].

*ROS-Extracellular Matrix (ECM)-IMMUNE Cell Loop.* The extracellular matrix (ECM) is a complex structural network of fibrous proteins, proteoglycans, and glycoproteins. Aside from its role during the healing phase of mucositis, during which ECM signalling plays a critical role in establishing the fate of wound resolution, it also may have an active role during the more proximal aspects of radiation or chemotherapy-induced damage. A relationship between changes in ECM component expression and chemotherapy-induced intestinal injury was recently reported [141]. Furthermore, ECM mediates mesenchymal-epithelial communication and is also a reservoir of latent cytokines (such as TGF-*β* and IL1-*β*), which can be activated in consequence of the action of proteases (plasmin and thrombin) and ROS [142, 143]. Activation of MMPs through oxidants, which are generated by leukocytes or other cells, follows [144]. In turn, MMPs control chemokine activity [82]. Activated cytokines and chemokines, in turn, attract and activate other immune cells (such as neutrophils), which in turn, release ROS, thus restarting the loop.

*Endothelial-Epithelial Loop.* Morphologic evidence from histological studies using light and electron microscopy shows damage to the microvascular endothelial cells occurring earlier than that to the epithelium [83, 121]. Vascular injury involves microvessels and includes endothelial damage and coagulative occlusion [79]. Endothelial-cell apoptosis [84] interrupts the protective effect on the epithelia due to the endothelial-produced keratinocyte growth factor (KGF) [85], which activates the protective Nrf2-antioxidant pathway [48] in the epithelia. This results in epithelial thinning due to a loss of epithelial stem cells, which in turn generates further endothelial damage, ultimately leading to ulceration.

#### 2.2.2. Abscopal Effects and Toxicities: Systemic and Interorgan Signalling

**Figure 1:**
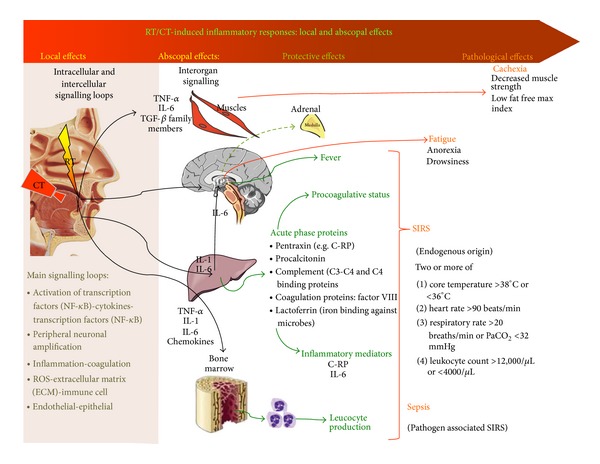
RT/CT-induced inflammatory responses: local and abscopal effects. Intracellular and intercellular signalling loops follow initiation and upregulation due to the local effects of chemoradiation on the exposed tissues. Released cytokines act not only locally but also on other organs and tissues (Interorgan signalling). On muscles they can alter energetic metabolism (thus favouring cachexia). On HPA (hypothalamic-pituitary-adrenal axis) they cause fever and fatigue symptoms. In the liver they provoke the synthesis of acute phase proteins that in turn act in a procoagulative and general inflammatory and antiinflammatory sense. All these effects can lead to systemic inflammatory response syndrome (SIRS) or sepsis. Abbreviations: PaCO_2_: arterial carbon dioxide tension. For other abbreviations, see the text.

The abscopal effect was the term that Mole proposed sixty years ago to describe the observation that patients demonstrate a range of responses distant from the radiated tumour [145]. Initially, abscopal effects were focused on distant tumour response, but it is now clear that the same phenomenon is relevant to normal tissue response and the aetiology of systemic side effects of treatment. In an excellent review of the topic, Siva et al. note that “localized irradiation perturbs the organism as a whole” [16]. They propose that the basis of the response stems from the development of a “chronic inflammatory environment and overall genomic instability.” The finding that focal radiation produces changes in gene expression detectable in peripheral blood monocytes consistent with pathways known to play a role in radiation toxicity [11] supports this hypothesis. The consequences of an abscopal effect may explain clinical observations of both “systemic” (fatigue, cognitive dysfunction, cachexia, etc.) and tissue centric (diarrhoea, nausea and vomiting, etc.) toxicities (Figure 1).

The two major mediators of the abscopal effect are cytokines and the immune system [16]. In healthy humans, cytokines are usually produced at low constitutive levels (picograms/mL) in plasma, and they function in an endo-, para-, or autocrine manner [146]. In inflamed (injured and/or infected) tissues, there is an excessive cytokine production that can become detectable in peripheral blood [11, 86, 147]. As noted above, a cytokine cascade is an established consequence of RT. The production of proinflammatory cytokines, particularly IL-1*β*, IL-6, and TNF-*α*, occurs not only in tissue, but in peripheral blood and increased levels of systemic proinflammatory cytokines which correlate with nonhaematological toxicities [121, 148–151] after RT, suggesting that the mediators of toxicities are not simply compartmentalised into the radiation field. Indeed, peripheral activated cells (such as B-lymphocytes [152], myeloid lineage cells [149], and monocytes [11]) have an increased transcription of inflammation-related genes, particularly those responsive to the proinflammatory NF-*κ*B transcription control pathway. In addition, it is conceivable that the use of concomitant CT offered an additional opportunity for the systemic effects to occur [11, 62, 153].

Cytokine production from activated cells is likely enhanced by activation of an innate immune response (see above) triggered by RT or CRT and tissue-borne pathogens. As a result peripheral blood levels of cytokines increase and remain elevated as has been reported in patients being treated for cancers of the head and neck [154]. A similar inflammatory response has been noted subsequent to CT-induced injury to the oral or gastrointestinal mucosal barrier where a systemic inflammatory response is elicited when various PRRs expressed by nonepithelial cells within the mucosa (e.g., macrophages, neutrophils, and dendritic cells) are exposed to severe endogenous (DAMP) and exogenous (PAMP) stress [32, 155–158]. For example, Blijlevens and colleagues reported that mucositis induced a systemic inflammatory response characterized by fever in neutropenic stem cell recipients, even in the absence of bacteraemia [156].

The impact of systemic cytokines as mediators of clusters of toxicities was suggested by Aprile et al., who used a Bayesian analytical approach in a cohort of patients receiving CT to treat colorectal cancer. They noted that some toxicities were more frequently interconnected than would be expected by chance [1]. The most frequently associated toxicities were those that probably share a common pathobiology (fever, fatigue, anorexia, and weight loss) [1]. Furthermore, some authors [2, 159] showed the plausibility of these associations, whereas others showed that it is possible to predict both the risk of oral mucositis [160] and severe sepsis [161, 162] by the dosage of plasma levels of inflammatory mediators in patients receiving mucotoxic treatments.

Thus, it is conceivable that the CT-RT-induced inflammation can act both locally, as a consequence of a paracrine signalling amplification, and systemically, as a consequence of a sort of endocrinal-like signalling amplification.

*Behavioural Examples of Abscopal Toxicities.* While behavioural toxicities such as fatigue and cognitive dysfunction are commonly recognised to occur among patients receiving CRT, it is only recently that the physiological basis for these changes has been assessed in the context of focal RT regimens. We know that the nervous system can be activated systemically by circulating cytokines, such as IL-6 [88]. The finding that the microvasculature in the mediobasal hypothalamus has a specialised fenestrated endothelium might explain the transfer of cytokines from the circulation to the central nervous system (CNS), where they in turn could stimulate the local production of cytokines [89] or initiate behavioural changes. Thus, while the sensory nervous system activates the sympathetic nervous system both segmentally, at the level of the spinal cord, and centrally, the circulating cytokines (such as IL-6) provide a sensor for the extent of inflammation and the consequent energy needed at a systemic level [161, 162]. Specifically, the hypothalamic-pituitary-adrenal (HPA) axis has been identified as a possible conduit for the mediation of behavioural toxicities. Concomitantly, parasympathetic activity, which has anti-inflammatory effects [163, 164], is inhibited during initial inflammation [165, 166] in order to modulate adequate inflammation intensity. At any rate, parasympathetic activity plays a role in systemic inflammation such as sepsis. Indeed the experimental stimulation of the peripheral vagus nerve strongly inhibits lipopolysaccharide-induced acute inflammation [167] and leukocyte recruitment [168].

To further support the link between CT-RT induced inflammation and the clinical symptoms frequently associated to mucositis, recent literature has shown that fatigue [90, 169], cachexia [94], and Systemic Inflammatory Response Syndrome (SIRS) [71, 169, 170] have been associated to a deregulated systemic inflammatory response to CT-RT of the organism (Figure 1).

*(a) Fatigue and Systemic Inflammation.* Fatigue is defined as the physical and/or mental weariness resulting from exertion: an inability to continue exercise at the same intensity with a resultant deterioration in performance [97, 171].

The underlying mechanism of cancer-related fatigue (CRF) remains unclear; it is probably due to multifactorial causes [172]. Prue's systematic review [173] found that there were significant increases in fatigue during anticancer therapy (fatigue prevalence 39%–90%).

Fatigue has been demonstrated to increase during RT [174, 175] and does not depend on concomitant increases in emotional distress [176, 177]. Yet two randomised controlled trials have reported that while paroxetine improves the mood of patients undergoing outpatient CT, it has no effect on fatigue relief [178, 179]. Taken together, these studies suggest that fatigue and depression/psychological distress are related but distinct phenomena. A number of authors have suggested that CRF may be related to an elevated or prolonged inflammatory response in cancer patients [180]. Two recent reviews showed a positive association between CRF and inflammatory/immunity circulating marker levels during RT and CT [90, 181]. However, the majority of studies was cross-sectional in nature and did not use well-validated fatigue assessment instruments.

*(b) Cachexia and Systemic Inflammation.* A new definition/classification [97, 98] of cancer cachexia has only recently been made (Table 1). Since no standard definition had been available, cachexia was previously underdiagnosed [182]. Even today, physicians usually treat the symptoms of anorexia, weight loss, and insulin resistance without diagnosing cachexia. In the retrospective study by Fox et al. [182], physicians diagnosed cachexia only in 6.1% of 246 patients, even though 19.9% of them had lost >5% of their body weight and 37% of them had at least one of the cachexia definitions.

Cancer cachexia has a multifactor pathogenesis due to tumour-releasing factors [183, 184] or to treatment [185]/sepsis immune responses, or both.

Indeed, the circulating inflammatory cytokines such as TNF-*α* [186, 187], IL-6, TGF*β* family members (e.g., myostatin and activin) seem to have a role in inducing cachexia acting both peripherally on the muscle and centrally on the CNS.

Peripherally, cytokines activate NF-*κ*B in muscles [188] and promote muscle degeneration by accelerating protein breakdown [91] and by dysregulating protein-turnover leading to catabolism [189].

Centrally, the cytokines act especially on HPA axis. Indeed, the rise in circulating cytokines has been implicated in the physiologic and behavioural responses to inflammation in rodents, including anorexia [190], HPA axis activation [191], and fever [192].

In humans, IL-6 [193] seems to have an important role. Recent trials on a monoclonal anti-IL-6 antibody used in order to contrast lung cancer patients' weight loss have shown that it is able to reverse anorexia, fatigue, and anaemia, but it had no significant effect on the lean body mass loss [194].

The study by Silver et al. [185], who treated seventeen HNCPs with induction CT followed by concomitant CT-RT, is interesting because it related wasting syndrome to CT-RT. These patients showed a wasting syndrome with a statistically lean body mass loss (*P* = .005), declined total physical activity, increased resting energy expenditure (*P* = .019), and increased inflammatory cytokines and inflammatory mediators (C-reactive protein (C-RP), *P* = .09 and IL-6, *P* = .08)) during concomitant CT-RT even though there were no significant differences in energy intake or calorie/nitrogen ratio from pretreatment to posttreatment.

Other authors have found that C-RP was an independent predictor of weight loss (*P* < .001) in HNCPs treated with CRT [195].

We hypothesise that in HNCPs, the cachexia syndrome is particularly reinforced by systemic inflammation induced by oral mucositis and reduced energy intake due to reduced swallowing capacity [195, 196].

## 3. Conclusions 

Much has been learned about the pathogenesis of cancer regimen-related toxicities. The complexity of the molecular and cellular response to chemotherapy and radiation, the observation of distant or systemic toxicities following focal radiation therapy, and the discovery of genomic features that are associated with toxicity risk have only found their way to mainstream thinking in the past decade.

It is now clear that the molecular events that occur within tissues following radiation begin within seconds of the challenge. And the consequences of the resulting biological cascade not only result in local tissue injury, but in the release of active proteins in the circulation. It is this systemic cytokine storm and its companion molecules that lead to abscopal effects in normal tissue. Clinically, these are manifest by both distant distal injury and by behavioural toxicities such as fatigue, cognitive dysfunction, and cachexia.

Furthermore, it is now clear that the clustering of toxicity symptoms that is most commonly not random. Rather, these changes, which distinct clinically represent the consequences of shared biology. It is the target tissue responding to common drivers that results in the distinct phenotypes. Thus, the concept of toxicity syndromes defined, not clinically, but by common bioetiological features is becoming increasingly important in the determination of patient status and in establishing pharmacological targets.

Ultimately, it is the discovery and definition of the pathways that lead to toxicities that will define and optimise a comprehensive approach to their amelioration.

## Figures and Tables

**Table 1:** Local and systemic pathogenesis and consequences of regimen-induced inflammatory responses.

Mucosal injury	Relevant clinical consequences
(1) Initiation: oxidative stress and the innate immune response	
Cellular damage induced by CT-RT (i) X-rays or mucotoxic drugs cause direct DNA break [28, 37]. (ii) Generation of ROS [38]. (iii) ROS damage lipids, DNA, connective tissue, and other biomolecules [39–41]. (iv) Cells die in epithelia, endothelia [62, 63], and submucosal tissue [28]. Release of inflammatory substances The components passively released from injured cells become a danger signal that alert the host of the dying cells and play an integral role in initiating toxicity [15]: (i) intracellular proinflammatory CRAMP (e.g., HMBG1 [64, 65], mitochondrial derived substances [40] ect.), (ii) intracellular enzymes (lysosomial), which activate extracellular proinflammatory DAMPs [66, 67] (which in turn activate other cascades, i.e., clotting, fibrinolytic, and kin cascades), (iii) altered redox state of the injured tissue [44], (iv) presynthesised interleukins (IL-1*α*, IL-33) [68–70], (v) released intracellular hidden antigens which activate Complement via antibodies (Complement can be regarded both as a PRR system and an effector system [71, 72]).	**Silent phase**

(2) Upregulation/activation	
(i) Activation of PRR, IL-1R, and RAGE receptors of the host's innate immune system [15, 53] and of the peripheral nociceptive nervous fibres [73, 74]. (ii) The main canonical pathways associated with the development of CT-RT mucositis [11] as follows: (1) nitrogen metabolism (2) TLR signalling (3) NF-*κ*B signalling (4) B cell receptor signalling (5) PI3K/AKT signalling (6) G2/M DNA damage checkpoint (7) SAPK/JNK signalling (8) P38 MAPK signalling (9) Wnt/B-catenin signalling (10) glutamate receptor signalling (11) integrin signalling (12) VEGF signalling (13) IL-6 signalling (14) death receptor signalling	**Inflammation: transient faint erythema and pruritus that can develop during the first hours after irradiation** [75, 76].

(3) Signal amplification and feedback	
Local effects: intracellular and intercellular signalling loops	
(i) Activation of Transcription factors (NF-*κ*B)—Cytokines (e.g., TNF-*α*)-Transcription factors (NF-*κ*B) loop [28] (ii) Peripheral neuronal amplification loop [77, 78] (iii) Inflammation-coagulation loop [79, 80] (iv) ROS—extracellular matrix (ECM)—immune cell loop [81, 82] (v) Endothelial-epithelial loop [83–85]	**Local inflammation**: **Oedema**, Cellular (mononuclear cells/macrophage and neutrophil) infiltration Epithelial thinning: **hypersensitiveness** Vasodilatation: **erythema**
Abscopal effects and toxicities: systemic and interorgan signalling [71]) (Figure 1)	
(i) Elevated serum levels of NF-*κ*B, TNF-*α*, IL-1 and IL-6 [86, 87] (ii) Genetic changes in peripheral blood monocytes [11]. (iii) Plasma cascades (Complement, coagulation, fibrinolytic, and kallikrein-kinin systems) [71] (iv) Acute phase proteins (Pentraxins—C-RP/SAP, Factor XII, Complement proteins ect.) [71] (v) HPA axis activated by circulating cytokines (IL-6) and by peripheral nervous system [88, 89]	**Altered body temperature and altered metabolisms** **Fatigue** [90] **Cachexia** [91–93]*. **SIRS** [94]

(4) Ulcerative/microbiological phase	
(i) Mucosal barrier injury (ii) Bacterial colonisation: increases follow, not precede, ulceration/MBI [95, 96] (iii) Microflora shifts due to CT, xerostomia, antibiotic use, and neutropenia. (iv) Microorganisms penetrate the disrupted mucosa and stimulate infiltrating macrophages to produce additional proinflammatory cytokines.	**Ulceration** **Colonisation** **Local infection.**

*Cachexia: weight loss > 5% or BMI < 20 plus decreased muscle strength, fatigue, anorexia, low lean mass index, and abnormal biochemistry (increased C-RP and IL-6 inflammatory markers, anaemia, and low serum albumin) [97, 98].

Abbreviations see the text.
